# Hemorphins—From Discovery to Functions and Pharmacology

**DOI:** 10.3390/molecules26133879

**Published:** 2021-06-25

**Authors:** Przemyslaw Mielczarek, Kinga Hartman, Anna Drabik, Hao-Yuan Hung, Eagle Yi-Kung Huang, Ewa Gibula-Tarlowska, Jolanta H. Kotlinska, Jerzy Silberring

**Affiliations:** 1 Faculty of Materials Science and Ceramics, AGH University of Science and Technology, 30-059 Krakow, Poland; kinga.piechura24@gmail.com (K.H.); drabik@agh.edu.pl (A.D.); jerzy.silberring@agh.edu.pl (J.S.); 2 Laboratory of Proteomics and Mass Spectrometry, Maj Institute of Pharmacology, Polish Academy of Sciences, 31-343 Krakow, Poland; 3 Department of Pharmacology, National Defense Medical Center, Taipei 11490, Taiwan; n19850908@yahoo.com.tw (H.-Y.H.); eyh58@mail.ndmctsgh.edu.tw (E.Y.-K.H.); 4 Graduate Institute of Medical Sciences, National Defense Medical Center, Taipei 11490, Taiwan; 5 Department of Pharmacy Practice, Tri-Service General Hospital, National Defense Medical Center, Taipei 11490, Taiwan; 6 Department of Pharmacology and Pharmacodynamics, Faculty of Pharmacy with Division of Medical Analytics, Medical University of Lublin, 20-093 Lublin, Poland; ewagibula@umlub.pl (E.G.-T.); jolanta.kotlinska@umlub.pl (J.H.K.)

**Keywords:** hemoglobin, hemorphins, analysis, proteolytic enzymes, sequencing, mass spectrometry, identification, pain, alcohol, opioids, receptors

## Abstract

During the last three decades, a variety of different studies on bioactive peptides that are opioid receptor ligands, have been carried out, with regard to their isolation and identification, as well as their molecular functions in living organisms. Thus, in this review, we would like to summarize the present state-of-the art concerning hemorphins, methodological aspects of their identification, and their potential role as therapeutic agents. We have collected and discussed articles describing hemorphins, from their discovery up until now, thus presenting a very wide spectrum of their characteristic and applications. One of the major assets of the present paper is a combination of analytical and pharmacological aspects of peptides described by a team who participated in the initial research on hemorphins. This review is, in part, focused on the analysis of endogenous opioid peptides in biological samples using advanced techniques, description of the identification of synthetic/endogenous hemorphins, their involvement in pharmacology, learning, pain and other function. Finally, the part regarding hemorphin analogues and their synthesis, has been added.

## 1. Introduction

Hemorphins are endogenous peptides that are also known as “non-classical” or “atypical” opioid peptides. They are produced under physiological [1] or pathological [2] (inflammation) states by hemoglobin proteolysis. They can be released from almost any of the hemoglobin chains (beta-, kappa-, delta-, or epsilon-chain) except the alpha chain [3]. Biochemical analysis has confirmed the presence of a substantial concentration of hemorphins in the human pituitary gland [4], bovine hypothalamus [5], bovine brain [1], adrenal glands [6], as well as other organs [7] and body fluids [8]. The high stability of these peptides in plasma or tissues and their wide distribution may suggest significant role of these peptides in various processes [3].

The shortest sequence of hemorphins maintaining its binding to opioid receptors is Tyr-Pro-Trp-Thr. First report, published by the A. Herz′s group, described identification of hemorphin-4 (H-4) and H-5 as they were released from bovine blood by gastrointestinal enzymes [9]. This work was performed nearly accidentally, during identification of closely eluted cytochrophin-4 and, at that time, only amino acid analysis and Edman degradation were available, but these techniques were sufficient for a complete identification of the in vitro released products.

Hemorphins can be produced in vitro by endogenous lysosomal proteases [10], pepsin [11], pancreatic elastase [12] or cathepsin D [13,14].

It is still uncertain which enzymes are responsible for the generation of hemorphins from hemoglobin chains. It has been postulated that these peptides may also be released from other, hitherto unknown, proteins [3]. A recent, thorough search for the sequence of LVV-H-7 by the UniProtKB 2020_06 knowledgebase shows unequivocally that hemorphins are, indeed, derived from hemoglobin chains.

Opioid properties of several peptides, including hemorphins, beta-casomorphins and cytochrophins, were described by Zadina et al. [15] as showing their ability to inhibit binding of the brain peptide Tyr-MIF-1 (Tyr-Pro-Leu-Gly-NH2) to its high affinity sites in rat brain. In general, all hemorphin fragments bind to the mu-opioid receptor. However, various sequences may also bind to other types of receptors, such as delta or sigma sites, though to a lower extent. Further studies have indicated that these peptides may also maintain a balance between opiate and antiopiate activities. The peptides mentioned above were artificially generated from hemoglobin. The first report on the naturally occurring peptide was presented by Glamsta et al. [8].

Particular focus has been aimed at the identification of endogenous LVV-H7 in body fluids. Under physiological conditions, LVV-H7 is not detectable in human cerebrospinal fluid (hCSF). In contrast, cerebral hemorrhage triggers the release of this peptide to a very high level in CSF (estimated at 115–300 pmol/mL). This observation has led to the isolation and identification of this sequence by the gas-phase sequencing and also by direct sequencing by mass spectrometry [8], and also without extensive preseparation [16].

Here, we must also underline the multiple role of hemoglobin in the body, which arises from such studies. Major functions of the protein are oxygen transport and removal of carbon dioxide. Other roles of hemoglobin, discovered much later, are based on the release of opioid peptides—hemorphins and longer sequences—hemocidins, possessing antibacterial properties [17]. The latter, however, is out of the scope of this review.

The aim of this paper is to describe the discovery of endogenous hemorphins, to summarize techniques necessary for identifying and quantitating these peptides and to recapitulate pharmacology of these fragments in spite of potential application of synthetic analogs in therapy [18]. This review describes the entire history of studies on hemorphins and discusses this issue very broadly, by presenting an overview of the complete knowledge related to this group of peptides. We are aware of the fact that there are other review articles concerning hemorphins published during the last five years, but they were concerning highly specialized aspects of knowledge. This involves very good reviews published in 2016: hemorphins in cancer biology [19], hemorphins in multiple sclerosis, metabolic and immune aspects [20]; in 2018: the role of hemorphins in moods modulation and emotions [21], therapeutic effects of hemorphins in human and animal models [18]; and the latest review published in 2021 that focuses on pharmacological and functional targeting of G protein-coupled receptors (GPCRs) by hemorphins and their implication in physiology and pathophysiology [22].

## 2. Proteolysis of Hemoglobin β Chain

**Figure 1:**
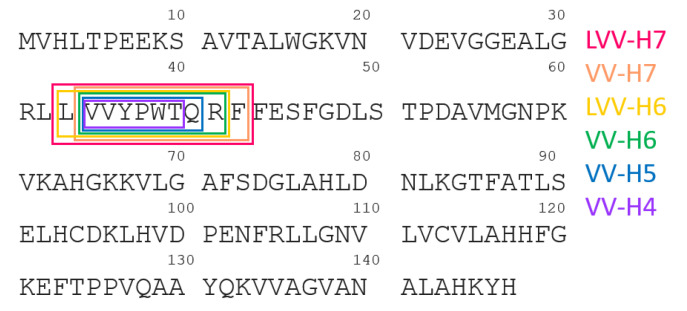
Scheme representing the release of opioid peptides from hemoglobin β chain.

There are several studies on the subject of sequential proteolytic digestion of hemoglobin β chain using various enzymes under different pH conditions. The common feature of those structures is the central core represented by the amino acid sequence: YPWT, see Figure 1 and Table 1. Among all presented opioid peptides, particularly LVV-H7 and VV-H7 are considered as the most stable forms. The first attempt to mimic the process of generation of hemorphins in an in vitro environment was originated by applying gastrointestinal enzymes [9]. However, Glamsta et al. pointed out the fundamental role of trypsin- and chymotrypsin-like enzymes in the formation of hemorphins [8]. The following experiments engaging pepsin and cathepsin D were described during research on the release of hemorphins form hemoglobin β chain [10,14,23]. The formation of V-H5 and H5 was observed after their release by pancreatic elastase [12]. Moreover, studies on the influence of pH conditions on the proteolytic process has led to the final conclusion that regulation of pH enables control of the degradation mechanism of hemorphins [24].

## 3. How to Identify Endogenous Molecule?

There is little information in the literature on a general strategy for identification of molecules of endogenous origin. Such compounds are being discovered for several reasons: (i) scientific curiosity; (ii) to reveal molecular mechanisms of a particular disease; (iii) to find a lead compound to design novel drugs. One of the giants in this field was Viktor Mutt (1923–1998) who identified a vast number of gastrointestinal peptides [25]. As the techniques were quite insensitive at his time, a substantial amount of biological material was necessary to fulfil the task. For this reason, almost all slaughterhouses in Sweden were collecting pig intestines for this research, and one kilometer of intestines resulted in ca. 1 milligram of pure hormone!

It is obvious that each molecule requires individual isolation protocols, and that the initial step-selection of the source and extraction procedure are crucial for further success of identification. There are, however, common rules that need to be obeyed as a gold standard. Here, we will focus on neuropeptides derived from the central nervous system, as their content is usually very low. Under specific circumstances, their level may be raised from undetectable to very high. A good example might be defensins upon inflammation and so is the case with hemorphins in cerebral bleeding. Such conditions, despite serious health problems, make it easier to purify and identify a given molecule. How can the knowledge on novel endogenous peptides be utilized? Neuropeptides reflect changes in their precursors, often containing other bioactive sequences. These processes are controlled by proteolytic enzymes and their inhibitors, which can also be of importance in pathophysiology. All these molecules can also serve as potential biomarkers in various diseases.

**Figure 2:**
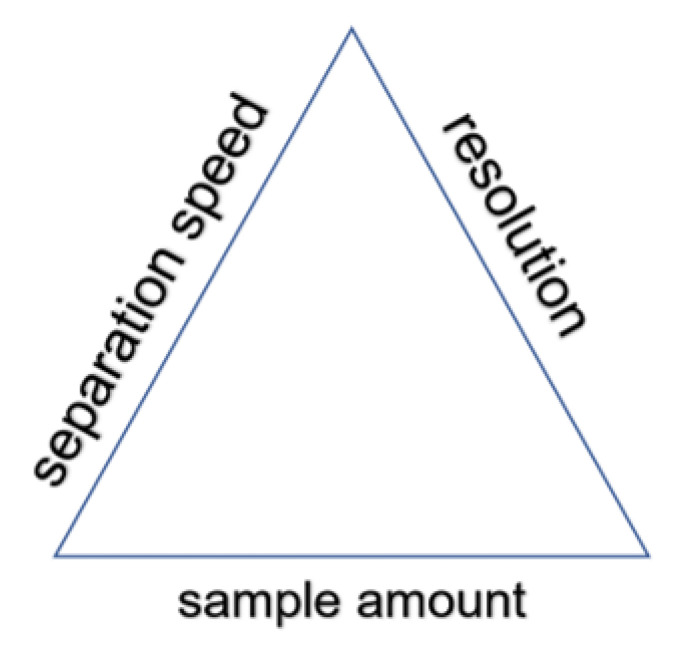
Separation triangle. Modification of any of the parameters is followed by changes of two other.

A good starting point might be solving a dilemma: sample amount, resolution or separation speed, as shown in Figure 2.

The proper answer will be substantially helpful in all following steps, and the important issue is collaboration with all parties already involved at the planning phase. This will avoid potential conflicting situations and a waste of time in solving the unresolved: “*whom to blame*”.

**Figure 3:**
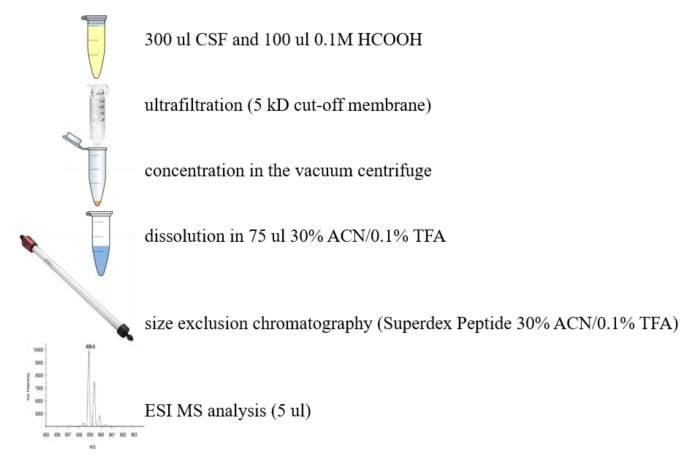
Isolation and identification of LVV-H-7 obtained from hCSF of patients suffering from cerebral bleeding, for direct MS analysis.

Nowadays, techniques became much more sophisticated and only a minute fraction of biological material is needed to isolate and identify a given molecule. A similar challenge was isolation and identification of endogenous hemorphins. The amount of biological material, i.e., human cerebrospinal fluid from patients suffering from cerebral bleeding, was available in sufficient quantities. A sample size of few hundred microliters of hCSF was necessary for the analysis and only limited preparation steps were applied as shown in Figure 3.

The above procedure led to the direct analysis of LVV-H-7 [16].

Molecular mass of a peptide (or any other molecule) is insufficient for its complete identification. Therefore, a similar approach, including fragmentation, has been used to reveal the sequence of endogenous LVV-H-7 in the same fluid [26]. After the reversed-phase chromatography, the sample was subjected either to a limited C-terminal sequencing by carboxypeptidase, or to N-terminal derivatization with a mixture of acetic anhydride/deuterated acetic anhydride (1:1 *v*/*v*). The first method allowed three C-terminal residues to be identified, and the latter approach provided complete sequence.

The discovery of hemorphins and their involvement in many biomolecular mechanisms was directly related to the design of proper isolation and purification methods. For the first time, the isolation of hemoglobin-derived opioid peptides form the CNS tissue was presented during studies on hemorphins′ activity in human pituitaries [4]. This research primarily enabled the identification of the absolute value of hemorphins using mass spectrometry-based approach. Following the elution in 1M acetic acid using Sephadex G-59 column, opioid peptides were isolated from CSF on the C-18 column and ACN/0.04%TFA gradient.

This methodology has also been used by others, thus allowing for the identification of other members of the hemorphins family in various rat tissues [7]. It was also applied for the examination of the level of opioid peptides extracted from dialysis membrane after hemofiltration of schizophrenic patients [27].

A modified scheme for hemorphin isolation was used during experiments described by Cohen et al. [28]. Hemoglobin hydrolysates were separated on the C-18 column and ammonium acetate buffer/ACN gradient, followed by the antibody-based VV-H7 identification.

Another option that enables the isolation and de novo sequencing of hemorphins in biological samples is based on the Sep-Pak C-18 cartridges with elution in ACN/0.1%TFA gradient followed by C-18 separation [29]. This work also discusses proteolytic degradation of opioid peptides by cathepsin D.

An alternative procedure for hemorphins isolation from the brain tissue was presented by Murillo et al. [30]. In this case, ultracentrifugation leading to the enrichment of subcellular fraction, followed by C-18 separation in ammonium acetate buffer/ACN and LC-MS analysis were applied. The half-lives of LVV-H7, V-H7 and H7 in protein homogenates form rat brain were tested, pointing out LVV-H7 as the most stable form of the hemorphins family.

Another procedure for LVV-H7 isolation from the animal blood as a food processing waste was described by Elagli et al. [31]. They presented a method allowing for continuous extraction of LVV-H7 with octan-1ol in a microfluidic reactor, followed by pepsin-based hemoglobin hydrolysis. Additionally, identification and isolation of opioid peptides from food protein derivatives was described by Teschemacher [32]. Furthermore, continuous production of LVV-H7 from bovine hemoglobin in a continuous membrane reactor was also reported by the group of Kapel et al. [33]. The proposed continuous-flow reactions allow for hemorphin extraction from animal blood that is a significant waste product of the food industry, thus simultaneously saving solvents and obeying environment preservation.

## 4. Identification and Quantification of Hemorphins

There are several instrumentation techniques used in hemorphins analysis, as well as techniques used in research experiments devoted to the study of their interactions in human body. The most commonly used approaches for identification of endogenous hemorphins in tissues are antibody-based methods and mass spectrometry techniques linked to liquid chromatography (LC-MS) or capillary electrophoresis (CE-MS), because of the high sensitivity and low limit of detection. Instrumentation used for characterization of synthetic products utilizes preparative chromatography and nuclear magnetic resonance (NMR), because higher amounts of the material can be analyzed. Interactions of hemorphins with proteins are studied by gel electrophoresis, fluorescence resonance energy transfer (FRET), bioluminescence resonance energy transfer (BRET) and molecular docking. For more details on these topics, see the following sections.

### 4.1. Endogenous Peptides

#### 4.1.1. Antibody-based Methods

Originally, H-7 was quantitated in human blood plasma, following long-distance running [34]. Radioimmunoassay was used for this purpose with the rabbit-raised specific antibodies. It is interesting that it was possible to achieve high specificity of the assay toward just H-7, whereas cross-reactivities for LVV-H6, H-6, H-4, hemoglobin were almost neglectable. A similar approach has been used for the measurement of H-7 in human cerebrospinal fluid [35]. It should be pointed out here that such immuno-based techniques always detect an “immunoreactive-like material”, even though several sample pre-separation steps are applied.

Nowadays, ELISA kits that are more suitable for the fast determination of hundreds of samples can easily be purchased from various vendors and are used for quantitation of hemorphins [36], but it should always be kept in mind that these techniques will provide general information on the “immunoreactive-like material”, despite characteristics of the antibody specificity attached to the kit, and that coelution with synthetic standard does not always reflect the real content under chromatographic peak when dealing with complex, endogenous material.

#### 4.1.2. Mass Spectrometry

Mass spectrometry still remains the main analytical technique involved in peptide identification, including hemorphins. Electrospray ionization (ESI-MS) is predominant in this respect. It was applied to identify hemorphins in many biological samples, such as mouse brain [37]. Additionally, ESI-MS, combined with liquid chromatography (LC-ESI-MS), was used to isolate hemorphin and hemorphin-like peptides presenting bradykinin potentiating activity from dog pancreas and sheep brain. This work by Ianzer et al. also investigates biological effect of LVV-H7 on blood pressure of the anaesthetized rats [29]. Liquid chromatography with mass spectrometry detection was also applied to clarify the question regarding whether hemorphins can cross the blood-brain barrier. This experiment was performed by Domender et al. who showed that all hemorphins, except LLVV-H4, are able to cross, in an intact form, the human intestinal epithelium model with Caco-2 cells, and all hemorphins can cross the human blood-brain barrier model with the brain-like endothelial cells [38]. LC-ESI-MS instrumentation was also helpful in identifying LVV-H7 in lipoaspirate fluid, in which proteomic components have been analyzed by the bottom-up and top-down approaches [39]. A huge number of hemorphins was identified in a single proteomic experiment, what was demonstrated by Iavarone et al. [40]. To emphasize the universality of this method, it should be mentioned that this paper described the most studied cryptides released from various sources, such as serum albumin, immunoglobulins, hemoglobin, saliva and milk proteins, thus showing ubiquitous nature of cryptic sequences [41]. Lately, based on LC-ESI-MS analyses, LVV-H7 was also reported to be present in gingival crevicular fluid obtained from the teeth from children [42].

Mass spectrometry was not only applied to identify hemorphins, but also to analyze their degradation and metabolism. Hayakari et al. presented that LVV-H7 is sequentially hydrolyzed by the angiotensin-converting enzyme from rat brain, leading to the release of LVV-H5. The experiment was performed with an application of liquid chromatography combined with frit-fast atom bombardment mass spectrometry and providing detailed degradation studies of hemorphins, also showing interesting substrate preferences during ACE action. Bearing in mind publication date, the technique used is somewhat unique; however, it was fully adequate for the tasks undertaken [43]. Later on, Murillo et al. performed identification of the degradation products by LC-ESI-MS methodology, resulting from the incubation of hemorphins, such as LVV-H7, VV-H7 and H-7, with subcellular fractions that were isolated from the rat brain, mainly leading to the release of LVV-H5 [30].

Mass spectrometry with electrospray ionization (ESI-MS) is not the only type of mass spectrometry applied for studies involving LVV-H7. John et al. investigated LVV-H7 digestion by aminopeptidase M identified as an LVV-H7 degrading enzyme, which is potentially involved in the regulation of LVV-H7 activity towards insulin-regulated aminopeptidase. In this experiment, quantitative detection of peptides was performed by the matrix-assisted laser desorpion/ionization time-of-flight mass spectrometry (MALDI-TOF-MS) [44]. In another study, John et al. also used LVV-H7 as an internal standard for quantification of peptides by MALDI-MS [45]. A mass spectrometry technique called surface-enhanced laser desorption/ionization time-of-flight mass spectrometry (SELDI-TOF-MS) was applied to identify LVV-H7 in intraluminal thrombus samples [46]. Based on this experiment, Dejouvencel et al. suggested that hemorphins might be used as a biological marker of pathological vascular remodeling. Matrix-assisted laser desorption ionization time-of-flight mass spectrometry (MALDI-TOF MS) was also applied for such purpose by Bucknall et al. [47] who demonstrated the practical utility of the MALDI-TOF MS for the unambiguous identification of molecules, but recommended quantitation by other means, as this ionization type is not suitable as a truly quantitative tool. Instead, e.g., ESI MS/MS can be used in such cases.

As demonstrated by Zeccola et al., capillary electrophoresis coupled with mass spectrometry (CE-MS) can be a powerful tool for hemorphins (LVV-H7 and VV-H7) analysis in cerebrospinal fluid [48]. The presented method allows for a reduced and rapid sample preparation, and was successfully applied in patient samples without matrix interferences arising from the complex biological sample. This was not for first time that capillary electrophoresis (CE) was used for the identification of hemorphins. In 2006, John et al. published an article presenting LVV-H7 and identification of its N-terminal degradation products in human plasma samples with the application of a multi-component capillary zone electrophoresis (CZE) [49]. The detection was based on the UV absorption instead of mass spectrometry, as it was described earlier. Later on, the same research group demonstrated suitability of CZE technique for the analysis of an in vitro degradation of LVV-H7 in mammalian plasma [50]. Finally, they also explored CZE to analyze subsequent in vitro proteolysis of LVV-, VV- and V-H7 by aminopeptidase M (AP-M), also coined as LVV-H7 degrading enzyme [44].

#### 4.1.3. Other Techniques

Polyacrylamide gel electrophoresis (PAGE) is usually applied for the separation of proteins and is not dedicated for small peptides. For this reason, PAGE is not used for hemorphin identification, but instead, for the analysis of its interactions with proteins. For example, such an experiment was performed by Barkhudaryan et al. using the two-dimensional fluorescence difference gel electrophoresis (2-D DIGE) and the isotope coded protein label technology (ICPL). Based on this methodology, peptidyl-prolyl cis-trans-isomerase A (cyclophilin A) was identified to be regulated by hemorphins in the mouse brain [51].

Fluorescence resonance energy transfer (FRET) is a technique providing evaluation of the interactions between a donor molecule, which is in the excited state, and an acceptor molecule, which is in its ground state. It is known that the energy absorbed by Tyr can be transferred to Trp. Todorov et al. synthetized new a azobenzene-containing VV–H5 containing -Tyr-Pro-Trp- peptide motif, and FRET was applied to study conformational changes of the peptide [52]. The main drawback of FRET is the requirement of external illumination to initiate the FRET. To overcome this limitation, bioluminescence resonance energy transfer (BRET) was introduced.

BRET was applied by Ali et al. [53] to study the effects of LVV-H7 on the angiotensin II type 1 receptor (AT1R) expressed in human embryonic kidney cells (HEK293). The expressed receptor was the GPCR-type complex, involving recruitment of beta-arrestin upon stimulation, followed by the ligand binding, and an interesting conclusion was drawn concerning the role of hemorphins in analgesia. This group also examined the binding behavior of LVV-H7 to AT1R and its effect on angiotensin II binding using nanoluciferase-based bioluminescence resonance energy transfer (NanoBRET) in HEK293FT cells [54].

Ali et al. presented computational methods to elucidate the alignment of mammalian LVV-H7 to mu-opioid receptors (MOR), angiotensin-converting enzyme (ACE) and insulin-regulated aminopeptidase (IRAP) that are its binding counterparts, and to calculate the binding affinity to these proteins [55]. It was shown that camel LVV-H7 produced stronger interactions with all studied proteins (MOR, ACE and IRAP) than non-camel LVV-H7. A year later, Ali et al. presented application of molecular docking to investigate binding of LVV-H7 to AT1R receptor [54]. It was reported that the performed interaction indicates that docking mechanism of LVV-H7 to angiotensin II type 1 receptor (AT1R) allosterically potentiates angiotensin II binding.

### 4.2. Synthetic Hemorphins

Nuclear magnetic resonance (NMR) requires a substantial amount of the sample for the reliable measurement, and therefore this method is not used for hemorphin identification in biological samples. However, it is one of the best analytical techniques for molecular characterization after chemical synthesis. Based on this, both 1H NMR and 13C NMR were performed for the characterization of solid-phase peptide synthesis (SPPS) products, in this case the azobenzene-containing VV-H5 [52].

As it was described in the previous section, in most experiments on hemorphins, chromatography is performed in connection with mass spectrometry detection. However, in some cases, chromatography can be used as an analytical method itself. Ianzer et al. used the semi-preparative chromatographic system for identification of several hemorphin-like peptides presenting bradykinin potentiating activity [29]. The eluted peaks were collected as separate fractions and later on analyzed by mass spectrometry. However, there were also studies that did not include mass spectrometry detection, where peptide identification was performed based on UV absorption spectra and retention times alignment. Fruitier-Arnaudin et al. demonstrated this methodology as a powerful tool in LVV-hemorphin-7 metabolism by renal cytosol and purified prolyl endopeptidase analysis [56]. It should be stressed here that such an approach does not provide unambiguous identification of the complete sequence when applied to the molecules of endogenous origin. It is, however fully suitable to study synthetic compounds and their fragments.

## 5. Pharmacology of Hemorphins

Various pharmacological effects of hemorphins are related to their high affinity to μ -, δ – and κ – opioid receptors [3,4], angiotensin (Ang) IV (AT4) receptor, bombesin subtype 3 receptor (hBRS-3) [57] or corticotropin releasing factor (CRF) receptors [58]. However, the binding of hemorphins to opioid receptors is much lower than that to classical opioid peptides. Simultaneously, some studies suggest that these peptides may affect the opioid receptor system in a manner similar to classic opiates, or endogenous opioid peptides (enkephalins and endorphins), due to the relatively high content of these hemoglobin fragments in the tissues [1,59].

Numerous functional interactions between hemorphins and β-endorphin, growth hormone, prolactin [3], substance P (SP) [2], neuropeptide Y, Met-ENK-Arg-Phe [60] or CRF [58,61] have been observed. It should be emphasized that hemorphins, as members of the endogenous protective system of the organism, play a significant role mainly in response to pathophysiological conditions (e.g., stress, inflammation, cancer). In this case, hemorphins, similar to other pleiotropic neuropeptides [62], serve as one of the homeostatic factors that activate compensatory systems within the organism. This effect could be observed as the occurrence of interactions between CRP, opioids and hemorphins in the hypothalamus [5] and pituitary gland [4] in response to stressful conditions [3].

## 6. Learning

Several behavioral studies have confirmed the beneficial effects of LVV-H7 on learning and memory. These effects were observed as improvements in spatial and conditioned learning and were manifested as avoidance memory in normal rats [63]. Moreover, it has been shown that hemorphins and other AT4 receptor agonists also reversed memory impairments caused by the administration of scopolamine and mecamylamine [64,65], ischemic injury [66], alcohol abuse [67] and bilateral perforant pathway lesions [68]. Similar effects of LVV-H7 and Ang IV on memory and learning were also observed [63,69], such as enhancement of spatial working memory in the plus maze spontaneous alternation task [70,71] or spatial learning in the Barnes maze task [63]. The similarity of action is probably related to the fact that both Ang IV and LVV-H7 are endogenous ligands of the AT4 receptor [72].

Indeed, this receptor has been found in appreciable levels throughout the brain, but is particularly concentrated in the regions involved in cognition. Moreover, later studies have clearly indicated that AT4 receptor is the transmembrane enzyme, insulin-regulated membrane aminopeptidase (IRAP), the inhibition of which leads to the facilitation of learning and memory [73]. Additionally, the comparable effects involving LVV-H7 and IRAP inhibitors [74,75] suggest that it may be the main mechanism of the procognitive action of hemorphins. This hypothesis is additionally supported by the observations that LVV-H7, unlike angiotensin, exerts no affinity for the AT1 receptor, but nevertheless enhances spatial working memory [70], spatial reference memory [63,69] and passive avoidance memory [69].

Several researchers suggest that the procognitive effects of hemorphins are due to their ability to enhance long-term potentiation of the rat hippocampus in vitro and in vivo [76,77]. This opinion is proven by the fact that these substances facilitate memory in the hippocampus-dependent tasks. Moreover, enhanced spatial working memory was observed after single administration of LVV-H7 in the plus maze spontaneous alternation task [70] and Barnes maze task [63]. However, additional research must be undertaken to determine the exact mechanism underlying these effects, as their recognition may provide a new cognitive repair strategy.

## 7. Pain

Only few behavioral studies have been conducted to assess the antinociceptive activity of hemorphins that was first suggested via binding studies. For example, using different rodent models, Cheng and colleagues [78] explored the analgesic effect of LVV-H7. Their studies revealed that LVV-H7, after intrathecal administration, was capable of attenuation carrageenan-induced hyperalgesia at the spinal level, which could not be reversed by the co-administration of naloxone. This indicates that the effect does not come solely from the activation of opioid receptors. At the supraspinal level, they also found that LVV-H7 produced a significant anti-hyperalgesia effect that could be completely blocked by naloxone administration. This suggests that an opioid-dependent mechanism lies behind this effect. These results were then extended by further remarks that LVV-H7 could not affect the change of the paw volume during inflammation, when given intrathecally or intracerebroventricularly. Moreover, this peptide did not evoke any activity in the tail-flick test and paw edema test. However, one of the most striking findings was the prolonged duration of the anti-hyperalgesia exerted by LVV-H7, which could last up to 5–7 h. The authors hypothesized that this outcome may be a result of the activation of a system that could prevent formation of the inflammation-induced central sensitization at the spinal cord level. These data significantly expand the knowledge about the effects of hemorphins, suggesting that the anti-hyperalgesic activity of LVV-H7 may be due to the blockade of IRAP at the spinal level and activation of opioid receptors at the supraspinal level [78].

Furthermore, additional research by the same group led to the conclusion that the antihyperalgesic effect promoted by LVV-H7 in the paw withdrawal test may depend on the activation of the oxytocin receptor, since this effect was reverted in the presence of atosiban (an oxytocin receptor antagonist). Simultaneously, a significant antiallodynia effect of LVV-H7 was also shown in a model of neuropathic pain. However, intrathecal injection of LVV-H7 produced potent antiallodynia only in a group of male mice, which indicates a pronounced sex difference of this effect. Thus, authors emphasize gender differences as an important factor that ought to be noted in pain treatment [79].

Due to the difficulties in pain management in alcoholics, the role of LVV-H7 in these effects was likewise determined. Hung et al. [80] indicated that chronic exposure to ethanol and ethanol withdrawal has led to a quantitative alteration of LVV-H7, which may have an effect on nociception. These results suggest that the deterioration of anti-nociception induced by alcohol is correlated with the decreased level of LVV-H7, which might be due to the alcohol-induced anemia. Therefore, it is anticipated that supplementation of LVV-H7 or pepstatin during alcohol use and withdrawal could be helpful in preventing and treating hyperalgesia.

## 8. Other Effects

Central administration of LVV-H7 also produced anxiolytic-like effects in the four-plate anxiety model that were comparable with the clinically effective anxiolytic agent, chlordiazepoxide. Given that anxiety is often comorbid with depression, Beyer and colleagues [81] evaluated the potential antidepressant action of LVV-H7. So far, experiments in which a variety of doses and routes of administration were followed, have not shown that this peptide induces antidepressant-like effects. Moreover, da Cruz et al. [82] presented contradictory results in their studies on the antidepressant effect of LVV-H7 in the forced swim test. These researchers also concluded that an increased release of monoamines mediated by AT4 receptor activation may be the mechanism through which LVV-H7 evokes anxiolytic-like and antidepressant-like effects. Thus, the straightforward role of LVV-H7 in depression-like behavior is not clear. Therefore, an additional mechanism involved in the anti-depressant effect triggered by LVV-H7 may be related to activation of oxytocin receptors.

It has also been observed that LVV-H7 induces a dose dependent effect on locomotor activity of rats after intracerebroventricular (i.c.v.) administration. Moreover, at the doses that did not exert any effect on locomotor activity (0.5 and 1 μg, i.c.v.), LVV-H7 did not induce any rewarding effect in the conditioned place preference (CPP) test. However, at the dose of 20 μg, this peptide was found to enhance dopamine turnover in the mesocorticolimbic terminals. This effect may be due to the sedative effects observed after administration of LVV-H7 at high amounts and reduced locomotor activity in the CPP test [83].

Beyond the aforementioned experiments, studies involving nanodrolone suggest that LVV-H7 may not play a crucial role in the development of opioid dependence, or at least it is not involved in in the nandrolone effect upon morphine dependence when given simultaneously [83].

Published data also indicate the contribution of hemorphins to the pathophysiology of cancer, Alzheimer’s disease and diabetes [56,84,85,86]. In addition, it should be underscored that hemorphins exhibit anti-inflammatory and immunoregulatory properties [85,87]. Furthermore, hemorphins affect IL-2 biosynthesis and IL-2 receptor expression in lymphocytes [85] and appear to be both positive and negative regulators of T cell proliferation [84,88].

Hemorphins are also recognized for their role in the blood pressure control, as the positive modulation of the angiotensin II (AngII) type 1 receptor (AT1R) by LVV-H7 was described by Ali A et al. [53]. Other studies were performed to study the role of LVV-H7 in vascular and renal systems [46,54]. Consequently, LVV-H7, or its analogs, may be used as a therapeutic reagent for blood pressure control or as a biomarker of a vascular disease.

## 9. Hemorphin Analogues

**Figure 4:**
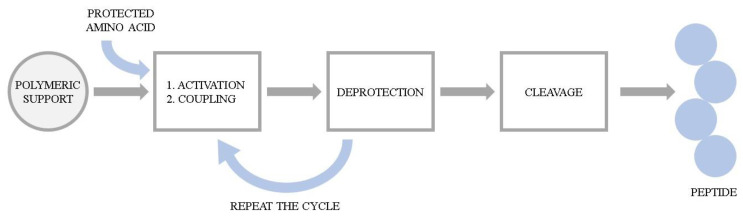
The general scheme of Fmoc solid phase peptide synthesis.

The series of hemorphin analogues have been synthesized and the structure–activity relationship of these compounds has been elucidated by Todorov’s group [89]. The synthesis of peptides was performed manually using 9-fluorenylmethoxycarbonyl (Fmoc) solid phase synthesis. The basic concept of the method is the stepwise construction of a peptide chain assembled on an insoluble polymeric support. Chain elongation starts at the C-terminal residue. The peptide formation goes through a repetitive amidation reaction between an activated carboxylic group of one amino acid and the amino group of the second one. Here, the coupling was accomplished via activation with 2-(1H-Benzotriazole-1-yl)-1,1,3,3-tetramethylaminium tetrafluoroborate (TBTU) and hydroxybenzotriazole (HOBt) in the presence of N,N-Diisopropylethylamine (DIEA). Each incoming amino acid was protected at the α-amino moiety by the Fmoc group to prevent formation of a peptide bond at that site. After coupling, the Fmoc group was removed using a 20% piperidine solution and the process was repeated. Due to the possibility of unintended reactions during synthesis, reactive side chains of the amino acids were also modified with the appropriate protecting groups. Upon completion of the chain assembly, the peptides were cleaved from the resin by simultaneous removal of the side chain-protecting groups. The cleavage of the synthesized peptides from the resin was performed using trifluoroacetic acid (TFA) with appropriate scavengers (phenol, triisopropylsilane (TIS), water). Subsequently, the compounds were precipitated from diethyl ether and dried under vacuum. The probes were further characterized by the high resolution electrospray ionization mass spectrometry and its purity was assessed by the preparative HPLC. The general scheme of Fmoc solid phase synthesis is presented in Figure 4.

To obtain the azobenzene-containing hemorphins, a solid-phase dimerization strategy was applied. This idea is based on the condensation of two units of the heptapeptide (VVYPWTQ) with the azobenzene moiety directly on the resin. Mainly, after formation of the heptapeptide (VVYPWTQ), the azobenzene-4,4′-dicarboxylic acid was coupled using TBTU/DIPEA and the coupling dimerization reaction was performed. Peptides were further cleaved from the resin, purified and analyzed according to the methods described above.

In the first set of experiments, C-amide peptide derivatives were prepared by incorporating non-proteinogenic/natural amino acids and azobenzene moiety in VVYPWTQ-NH_2_ (VV-hemorphin-5) and VVYPWTQRF-NH_2_ (VV-hemorphin-7) sequences. These analogues were further tested for visceral nociception using the writhing test in mice. The anticonvulsant activity of peptide derivatives was also evaluated in the three acute seizure tests; the maximal electroshock, the 6 Hz psychomotor seizure test, and the timed intravenous pentylenetetrazole infusion test. Concerning antinociceptive potency, the replacement of Gln position by Dap (2,3-diaminopropanoic acid) in VV-hemorphin-5 led to a derivative exhibiting the strongest analgesic effect among all peptides studied. It is noteworthy that, when isoleucine or 2-aminoisobutyric acid was added at position 1, a decrease in biological activity has been observed. Incorporation of Orn (2,5-diaminopentanoic acid) at Gln position of VV-hemorphin-5 and Dab (2,5-diaminobutanoic acid) moiety at Gln position of VV-hemorphin-7 peptide, respectively led to the analogues posessing good efficacy against the three seizure models [52,89,90,91,92].

VV-hemorphin-5 analogues modified at N-terminal with aminophosphonate group were characterized as potential anticonvulsants. Incorporation of (dimethoxyphosphyl)methyl)-leucine) moiety at two N-terminal Val positions allowed the most active derivative to be obtained [91]. To find an explanation of the possible molecular mechanism of action of the peptides with anticonvulsive properties, docking studies on delta- (DOR) and kappa- (KOR) opioid receptors have been elucidated. The results confirmed experimental data and suggested that the mechanism of the action of these peptides mostly involves activation of KORs.

Reports describing strong anticonvulsive and antinociceptive activities of several adamantane-modified molecules prompted Todorov’s group to perform the research in this direction. The new active analog of hemorphin-4 containing both adamantane and cyclohexane moieties have been synthesized [92]. Docking studies were performed using a model involving insulin-regulated aminopeptidase (IRAP), indicating that the obtained analogue is the most potent among tested inhibitors, and its biological activity has been further confirmed in the in vivo experiments.

Overall, design and synthesis of hemorphin analogues has led to significant changes both in the peptides′ activity and affinity towards opioids receptors or IRAP enzyme. Importantly, not only the position, but also the nature of the incorporated group had an impact on this phenomenon. The obtained data provide the basis for anticipation that the described compounds may be considered as a promising template for future design of the more active analogues, as well as in studies of other models of epilepsy [52,89,90,91,92,93].

Altogether, studies indicate that hemorphins comprise promising candidates for the therapy of pain, anxiety and depression or even hyperalgesia induced by ethanol withdrawal. However, the involvement of hemorphins in various pathological and physiological conditions requires future research.

## 10. Conclusions

To summarize, in this paper we have discussed different studies on hemorphins performed from the very beginning and over the last 30 years, with a focus on the recent findings in the field of opioid peptides. Moreover, this review describes unique strategies that were applied during the initial discovery of endogenous peptides. Today, hemorphins find their application as therapeutic agents or as biomarkers in various scientific disciplines, from neuropharmacology (pain, schizophrenia, depression, Alzheimer’s disease, and addiction treatment), to diagnostics (biomarkers of various disorders e.g., vascular and renal disease, cancer, inflammation), and finally, their pharmacological potential as future drugs. The presented studies on LVV-H7 distribution indicate a substantial role of opioid peptides in various pathological and physiological processes; however, the concentration of LVV-H7 and the conditions of their release form hemoglobin chains is not fully understood.

## Figures and Tables

**Table 1:** Amino acid sequences of hemorphins.

Hemorphin	Sequence
LVV-H7	LVVYPWTQRF
VV-H7	VVYPWTQRF
LVV-H6	LVVYPWTQR
VV-H6	VVYPWTQR
VV-H5	VVYPWTQ
VV-H4	VVYPWT
